# A journey from marginality to routine and beyond: single center experience with DCD utilization for liver transplantation in Italy

**DOI:** 10.1007/s13304-026-02581-2

**Published:** 2026-03-10

**Authors:** Guido Fallani, Alberto Stocco, Giorgia Radi, Enrico Prosperi, Antonio Siniscalchi, Maria Cristina Morelli, Matteo Cescon, Matteo Ravaioli

**Affiliations:** 1 Hepato-biliary and Transplant Surgery Unit, IRCCS Azienda Ospedaliero-Universitaria di Bologna, Via Massarenti, 9, 40138 Bologna, Italy; 2 Department of Medical and Surgical Sciences (DIMEC), University of Bologna, Bologna, Italy; 3 Department of Pharmacy and Biotechnologies (FABIT), University of Bologna, Bologna, Italy; 4 Postoperative and Abdominal Organ Transplant Intensive Care Unit, IRCCS Azienda Ospedaliero-Universitaria di Bologna, Bologna, Italy; 5 Internal Medicine Unit for the Treatment of Severe Organ Failure, IRCCS Azienda Ospedaliero-Universitaria di Bologna, Bologna, Italy

**Keywords:** DCD liver transplantation, Hypothermic oxygenated perfusion, Normothermic regional perfusion, Marginal donors, Extended criteria donors, Donation after cardiovascular determination of death

## Abstract

Donation after cardiovascular determination of death (DCD) has expanded the liver donor pool but remains limited by concerns over prolonged donor warm ischemia and inferior outcomes, particularly in countries such as Italy where legally mandated asystolic periods increase donor risk. This study evaluates trends in DCD liver transplantation at a high-volume Italian center, to assess whether accumulated experience and advanced reconditioning strategies influenced outcomes over time. We retrospectively analyzed adult DCD liver transplants performed between 2016 and 2023. Donor characteristics, recipient risk profiles, perfusion strategies, ischemia times, and post-transplant outcomes were compared between an early (2016–2021) and a late (2022–2023) period. Temporal trends were evaluated using linear regression. Seventy-five DCD liver transplants were included. Later-period recipients had more advanced liver disease (39.1% vs. 17.2% Child-Pugh C, *p* = 0.045) and higher P-SOFT and LTRS risk scores. Moreover, later-period donors were older (median 72 vs. 62 years, *p* = 0.022), and exhibited higher DRI and ET-DRI scores. Concurrently, HOPE duration increased, and total ischemia time decreased linearly with program maturation. Despite the progressive rise in donor and recipient risk, rates of early allograft dysfunction, biliary complications, retransplantations, and graft and patient survival remained unchanged between periods. In a high-risk regulatory setting, DCD liver transplantation outcomes remained stable despite increasing donor and recipient complexity. Program maturation, ischemia minimization, and prolonged hypothermic oxygenated perfusion appear to effectively mitigate DCD-associated risk, supporting broader and safer utilization of DCD liver grafts even in challenging clinical environments.

**Supplementary Information:**

The online version contains supplementary material available at 10.1007/s13304-026-02581-2.

## Introduction

Liver transplantation is considered the gold standard for the treatment of end-stage liver disease, and throughout the years it has been successfully applied for the treatment of various metabolic, congenital and oncological diseases. Over the last two decades the donor pool has expanded through the inclusion of donors with clinical and/or anthropometric characteristics associated with poorer outcomes (known as extended criteria donors, ECD) [1], allowing a reduction in waiting list drop-out and a progressive expansion of LT indications. Donors after cardiovascular determination of death (DCD) have also been utilized to increase the donor pool and have long been considered part of the ECD category due to the prolonged donor warm ischemia time (DWIT) inherent to cardiovascular determination of death and its detrimental effects on transplant outcomes. Indeed, early reports comparing DCD with donors after brainstem death (DBD) do show a net inferiority of DCD in terms of graft survival and biliary complications [2]. However, with increasing experience in DCD management and organ reconditioning strategies, the number of DCD donations has progressively increased, resulting in better outcomes in terms of ischemic time and retransplantation rates [3]. Furthermore, recent studies have reported comparable outcomes between DCD and DBD [4, 5], even in cases of DCD with extended criteria stigmata (EC-DCD) [6]. 

In the context of DCD, the Italian clinical setting is characterized by a prolonged asystolic period before death determination (20 min), whose obvious consequence is a long DWIT. For this reason, despite routine retrieval with normothermic regional perfusion (NRP) and end-ischemic hypothermic oxygenated perfusion (HOPE) [7], Italian DCDs have long been considered high-risk donors. Nevertheless, more recently they have been demonstrated to perform similarly both to non-Italian low-risk donors and to DBDs [6, 8]. 

This study examines the trends in DCD liver transplantation practices at a single high-volume transplant center in Italy, assessing whether DCD utilization and transplant outcomes have changed with the accumulated experience since the DCD liver transplantation program’s inception.

## Methods

We conducted a single center retrospective analysis of a prospectively collected database including consecutive adult recipients of liver transplants from Maastricht III-controlled DCD [9] from the beginning of the DCD liver transplant program at Policlinico Sant’Orsola, IRCCS Azienda Ospedaliero-Universitaria di Bologna, in January 2016, to December 2023. Recipients undergoing transplantation from donors procured with super-rapid recovery technique were excluded from the analysis (DCD donors in Italy can’t be procured with super-rapid recovery technique, but occasionally surplus donors from nearby countries are offered to Italian transplant centers).

The study aimed to assess post-transplant outcomes, donor profiles and recipient characteristics by comparing transplants performed in the initial years of the DCD liver transplant program (2016–2021) with the latest DCD transplants (2022–2023).

Informed consent for study enrollment was obtained from all the recipients; the requirement for informed consent is waived for the deceased donors. The study was conducted in accordance with the principles of the 1964 Helsinki Declaration and its following revisions and was approved by the Institutional Review Board of the promoting center (Comitato Etico – Area Vasta Emilia Centro, protocol n° 895/2021/Oss/AOUBo). The study and manuscript report comply with the Strengthening the Reporting of Observational Studies in Epidemiology (STROBE) guidelines [10]. 

### Technical aspects of procurement, graft perfusion and transplant

According to Italian legislation, donor death is determined following continuous electrocardiographic monitoring for 20 minutes, ensuring the absence of any cardiac electrical activity. Upon confirmation of death, the femoral vessels are promptly cannulated and connected to an extracorporeal support system. NRP is then initiated and maintained to evaluate lactate clearance, pH normalization, and liver function, with measurements recorded at the start of NRP and every 30 minutes thereafter. Once the metabolic profile and allograft function are deemed satisfactory, surgical retrieval is performed, and allograft biopsies are collected. At the conclusion of the procurement’s “warm phase”, NRP is discontinued, and the organs are flushed in situ with a cold perfusion solution (Celsior, Institut Georges Lopez – Lissieu, FR). Following extraction from the abdominal cavity, the liver allograft is stored in ice and transported to the transplant center.

End-ischemic hypothermic perfusion was conducted using the Vitasmart Hypothermic Oxygenated Machine Perfusion System (Bridge to Life Ltd. – Northbrook, IL) in all cases, beginning simultaneously with back-table graft preparation; perfusion was conducted through the portal vein only. During the initial back-table maneuvers, perfusion flow is set at 30 ml/min without perfusate recirculation, aiming to eliminate waste products and residual microthrombi from the allograft. Upon completion of back-table preparation, flow is increased to 250 ml/min with perfusate recirculation, ensuring continuous organ perfusion until implantation. Throughout all phases of HOPE, maximum perfusion pressure is maintained at 5 mmHg and oxygen is delivered to the membrane oxygenator with a 4 l/min flow. Detailed protocols regarding DCD donor management, organ viability criteria, procurement procedures, and pre-implantation reconditioning have been previously described [6, 11]. 

Organ allocation was established according to the Italian National Transplant Centre algorithms and the Emilia-Romagna Regional Transplant Center protocols [12]. The surgical team of the Department of Hepatobiliary and Transplant Surgery – Policlinico Sant’Orsola performed all retrieval procedures and transplants and managed all recipients in the post-operative period. Immunosuppression was managed with steroids and tacrolimus as per our center’s protocol [13]. Recipients’ biliary complications were investigated through magnetic resonance cholangiopancreatography in case of clinical suspicion and/or liver function tests alterations, and classified according to Esser et al. [14]

### Outcome definitions and measures

Functional DWIT (fDWIT) was defined as timeframe occurring between development of hypotension (mean arterial pressure below 60 mmHg) or desaturation (peripheral oxygen saturation below 70%) – whichever occurring first after withdrawal of life supporting treatment – and NRP initiation [15]. Ischemia time was defined as the interval from aortic cross-clamping and portal reperfusion upon LT, including both static cold storage (SCS) and HOPE duration.

Donor-related risk of graft loss was assessed using the Donor Risk Index (DRI) and the Eurotransplant Donor Risk Index (ET-DRI). To account for the smaller geographical scale of organ procurement in Italy compared with that of the original models, regional donations were classified as local, in accordance with the original scoring systems, and therefore did not receive additional points [16, 17]. Recipient’s risk of perioperative mortality and survival were measured with Liver Transplant Risk Score (LTRS) and Preallocation score to predict Survival Outcomes Following Liver Transplantation (P-SOFT) [18, 19]. The global risk of graft loss and recipient survival were assessed with SOFT score and UK DCD Risk Score [19, 20]. 

Donor categorization as ECD was established according to the criteria proposed by Vodkin et al. [21]

Complications were defined as any event deviating from the expected postoperative course, excluding sequelae inherent to the procedure, that did not imply failure to cure. For each patient, postoperative complications were graded individually according to the Clavien-Dindo classification [22] and summarized through the Comprehensive Complication Index^®^ (CCI^®^) [23]. 

Primary graft non-function (PGNF) was defined according to the Organ Procurement and Transplantation Network criteria [24], while early allograft dysfunction (EAD) was defined according to the criteria proposed by Olthoff et al. [25]

### Statistical analysis

Qualitative variables were reported as numbers (absolute values and percentages), whereas quantitative variables are reported as median values [interquartile range]. Univariate analysis was performed using Pearson’s chi-squared or Fisher’s exact test for categorical variables, depending on the sample size, and with Mann-Whitney’s U test for continuous variables. Univariate linear regression models tested whether donor and/or recipient risk scores as well as allograft reconditioning parameters changed with accumulated experience in DCD liver transplantation, using the transplant date as the independent variable.

The follow-up was censored at the end of December 2024.

Differences of *p* < 0.05 were considered significant.

All statistical analyses were performed using IBM SPSS, version 26.0 (IBM Corporation – Armonk, NY).

## Results

Between 2016 and 2023, a total of 84 controlled DCD liver allografts were offered for transplantation at the Department of Hepatobiliary Surgery and Transplantation, Policlinico Sant’Orsola, Bologna. Of these, eight were discarded, resulting in a final acceptance rate of 90.5%. Specifically, five allografts were rejected due to suboptimal metabolic and laboratory parameters during NRP, while the remaining three were discarded due to prolonged ischemia (> 12 h), extensive macrosteatosis, and incidental discovery of intrahepatic lithiasis during procurement, respectively. Among the 76 transplanted allografts, one was excluded from analysis as it was a surplus graft from Switzerland retrieved using super-rapid procurement technique. Consequently, the final study cohort comprised 75 liver allografts transplanted into 75 adult recipients.

The study population was stratified into two time periods: 2016–2021 and 2022–2023. During the initial period, 29 (38.7%) DCD liver transplants were performed, while 46 (61.3%) were conducted in the later period. Over the study timeframe, DCD liver transplants accounted for 9.2% of all liver transplants performed (*n* = 828), with annual rates increasing from 1.3% in 2018 to 18.5% in 2022 (Supplementary Fig. 1). DCD allograft acceptance rate showed a slight increase from 85.3% in the initial period to 93.9% in the later period (29/34 vs. 46/49, *p* = 0.192).

### Recipients’ characteristics

Recipients’ anthropometric data and indications for liver transplant were comparable between the two periods; notably, during the late era DCDs were utilized for urgent retransplantation in one case and combined liver-kidney transplantation in another case. Recipients’ clinical characteristics were also similar, except for a higher prevalence of Child-Turcotte-Pugh class C recipients in the later period (39.1% vs. 17.2%, *p* = 0.045). During the later period, recipients were characterized by higher LTRS (1 [0–2] vs. 1 [0–1], *p* = 0.014) and P-SOFT scores (6 [4–9] vs. 4 [2–7], *p* = 0.026). Recipients’ characteristics are summarized in Table 1.

**Table 1:** Recipients’ characteristics

Variables	Initial period (*n* = 29)	Later period (*n* = 46)	*p*
Recipient age in years, median [IQR]	59 [55–65]	60 [53–67]	0.752
Recipient BMI in kg/m^2^, median [IQR]	24.8 [23.2–28.9]	24.9 [22.5-27-6]	0.453
LT indication			0.419
HCC, n (%)	17 (58.6)	20 (43.5)	
Virus-related cirrhosis, n (%)	3 (10.3)	4 (8.7)	
ALD, n (%)	2 (6.9)	3 (6.5)	
MASLD, n (%)	0	4 (8.7)	
Cholestatic liver disease, n (%)	3 (10.3)	6 (13)	
Non-HCC malignancy, n (%)	4 (13.8)	4 (8.7)	
Polycystic liver disease, n (%)	0	4 (8.7)	
Retransplantation (HAT), n (%)	0	1 (2.2)	
Combined liver-kidney transplant, n (%)	0	1 (2.2)	1
Previous abdominal surgery, n (%)	17 (58.6)	23 (50)	0.466
Previous liver resection, n (%)	3 (10.3)	8 (17.4)	0.513
Recipient platelet count *10^9^/l, median [IQR]	88 [61–158]	115 [70–183]	0.409
MELD score, median [IQR]	12 [9–15]	12 [9–18]	0.698
MELD ≥ 25, n (%)	1 (3.4)	5 (10.9)	0.396
Portal thrombosis			0.732
Absent, n (%)	24 (82.8)	41 (89.1)	
Partial, n (%)	4 (13.8)	4 (8.7)	
Complete, n (%)	1 (3.4)	1 (2.2)	
TIPSS, n (%)	2 (6.9)	1 (2.2)	0.555
Child-Turcotte-Pugh class			
A, n (%)	15 (51.7)	16 (34.8)	0.147
B, n (%)	9 (31)	12 (26.1)	0.642
C, n (%)	5 (17.2)	13 (39.1)	**0.045**
LTRS, median [IQR]	1 [0–1]	1 [0–2]	**0.014**
P-SOFT score, median [IQR]	4 [2–7]	6 [4–9]	**0.026**

IQR,interquartile range; BMI, body mass index; HCC, hepatocellular carcinoma; ALD, alcohol-associated liver disease; MASLD, metaboplic dysfunction-associated steatotic liver disease; HAT, hepatic artery thrombosis; MELD, model for end-stage liver disease; TIPSS, transjugular intrahepatic porto-systemic shunt; LTRS, liver transplant risk score; P-SOFT, preallocation score - Survival outcomes following liver transplantation

### Donors’ characteristics and donation parameters

In the later period, donors had a significantly higher age (72 [58–79] vs. 62 [50–72] years, *p* = 0.022) and – excluding donation after cardiovascular determination of death – were most often characterized as ECD (95.7% vs. 75.9%, *p* = 0.023), with multiple criteria for ECD categorization other than DCD per single donor (2 [2–3] vs. 1 [0–2], *p* = 0.006). Causes for donor categorization as ECD (excluding donation after cardiovascular determination of death) across the two study periods are reported in Table 2.

**Table 2:** Donor’s characteristics and donation parameters

Variables	Initial period (*n* = 29)	Later period (*n* = 46)	*p*
Donor age in years, median [IQR]	62 [50–72]	72 [58–79]	**0.022**
Donor BMI in kg/m^2^, median [IQR]	24.7 [22.7–28.1]	25.8 [22.9–29.3]	0.617
Extended criteria donor (excluding DCD), n (%)	22 (75.9)	44 (95.7)	**0.023**
Age ≥ 65 years, n (%)	10 (37)	27 (58.7)	**0.041**
Macrovescicular steatosis > 30%, n (%)	0	1 (2.2)	0.424
ICU stay > 7 days, n (%)	10 (37)	25 (54.3)	0.093
Serum sodium > 165 meq/l, n (%)	0	1 (2.2)	0.424
Serum bilirubin > 3 mg/dl, n (%)	1 (3.4)	1 (2.2)	0.739
SGOT/SGPT > 3-fold URL, n (%)	3 (10.4)	11 (23.9)	0.142
Vasopressor use, n (%)	6 (20.8)	8 (17.4)	0.721
Potential for disease transmission to the recipient	1 (3.4)	3 (6.5)	0.564
Ischemia time > 12 h, n (%)	0	0	n.s.
Number of extended criteria other than DCD per donor, median [IQR]	1 [0–2]	2 [2–3]	**0.006**
DRI, median [IQR]	2.65 [2.35–2.92]	2.93 [2.51–3.15]	**0.010**
ET-DRI, median [IQR]	2.37 [2.08–2.64]	2.66 [2.32–2.93]	**0.012**
SOFT score			
Low risk, n (%)	11 (37.9)	17 (37)	0.932
Low-moderate risk, n (%)	15 (51.7)	24 (52.2)	0.970
High-moderate risk, n (%)	3 (10.4)	3 (6.5)	0.552
High risk, n (%)	0	2 (4.3)	0.255
UK DCD risk score			
Low risk, n (%)	1 (3.4)	1 (2.2)	0.687
High risk, n (%)	12 (41.4)	13 (28.3)	0.241
Futile, n (%)	16 (55.2)	32 (69.5)	0.206
Allograft microsteatosis in %, median [IQR]	0 [0–5]	2 [0–5]	0.223
Allograft macrosteatosis in %, median [IQR]	0 [0–5]	1 [0–5]	0.815
Allograft fibrosis			**0.041**
Ishak S0-S1, n (%)	19 (65.5)	19 (41.3)	
Ishak S2-S3, n (%)	10 (34.5)	27 (58.7)	
fDWIT in minutes, median [IQR]	43 [38–49]	40 [36–45]	0.160
NRP duration in minutes, median [IQR]	214 [182–244]	207 [172–227]	0.452
HOPE duration in minutes, median [IQR]	70 [60–137]	138 [115–155]	**< 0.001**

Bold highlights statistical significancy

IQR, interquartile range; BMI, body mass index; DRI, donor risk index; DCD, donation after cardiovascular determination of death; ICU, intensive care unit; SGOT, serum glutamic oxaloacetic transaminase; SGPT, serum glutamic pyruvate transaminase; URL, upper reference limit; ET-DRI, EuroTransplant donor risk index; SOFT, survival outcomes following liver Transplantation; DCD, donors after cardiovascular determination of death; fDWIT, functional donor warm ischemia time; NRP, normothermic regional perfusion; HOPE, hypothermic oxygenated perfusion

Moreover, donors in the later period had significantly higher DRI and ET-DRI scores (2.65 [2.35–2.92] vs. 2.93 [2.51–3.15], *p* = 0.010 and 2.37 [2.08–2.64] vs. 2.66 [2.32–2.93], *p* = 0.012, respectively). fDWIT and NRP duration were comparable between periods, while HOPE duration significantly increased in the later period (138 [115–155] vs. 70 [60–137] minutes, *p* < 0.001). Micro- and macrosteatosis percentages from pre-allocation biopsies were similar in both periods, though grafts with S2 Ishak portal fibrosis were more commonly utilized in the later period (54.3% vs. 31%, *p* = 0.048). Donors’ characteristics and donation parameters are summarized in Table 2.

### Intra- and postoperative outcomes

In the later period, allografts experienced shorter ischemia times (341 [315–375] vs. 390 [355–442] minutes, *p* = 0.002), and recipients had a lower peak serum glutamic-pyruvic transaminase (SGPT) value (398 [154–777] vs. 626 [311–1484] U/l, *p* = 0.018). Post-operative outcomes were comparable between periods, with similar ICU and hospital stays, as well as surgical complication rates. EAD, hepatic artery thrombosis (HAT), PGNF and retransplant rates were also similar between periods.

During the initial period three patients experienced PGNF, leading to retransplantation in two cases and patient death in one case; a third retransplantation was performed for prolonged graft dysfunction. In the later period, three retransplantations were performed: one for HAT, one for PGNF and one for prolonged graft dysfunction. Another HAT case was treated with surgical thrombectomy. Three early (< 1 year) graft losses due to patient death were observed in the initial period: one related to graft dysfunction and two to causes unrelated to graft function (cerebral hemorrhage and necrotizing pancreatitis). In the later period, two patients died within the first year due to graft dysfunction, while three additional patients died from causes unrelated to graft function (post-transplant lymphoproliferative disorder in one case, intraoperative refractory cardiac arrest in one case, and cerebral hemorrhage in one case). One-year biliary complication rates were comparable between the two cohorts (10.3% vs. 10.9%, *p* = 0.943), with no patients developing non-anastomotic strictures in either group. Intra- and post-operative outcomes are summarized in Table 3.

**Table 3:** Intra- and post-operative outcomes

Variables	Initial period (*n* = 29)	Later period (*n* = 46)	*p*
Operative time in minutes, median [IQR]	480 [384–508]	425 [386–474]	0.183
Ischemia time in minutes, median [IQR]	390 [355–442]	341 [315–375]	**0.002**
SGOT peak up to POD7 in U/l, median [IQR]	749 [317–1607]	464 [234–1057]	0.124
SGPT peak up to POD7 in U/l, median [IQR]	626 [311–1484]	398 [154–777]	**0.018**
EAD, n (%)	4 (13.8)	2 (4.3)	0.198
HAT, n (%)	0	2 (4.3)	0.255
PGNF, n (%)	3 (10.3)	1 (2.2)	0.293
Retransplant, n (%)	3 (10.3)	3 (6.5)	0.552
Clavien-Dindo > 2, n (%)	6 (20.7)	12 (26.1)	0.594
Comprehensive Complication Index^®^, median [IQR]	20.9 [8.7–34.8]	29.6 [17.9–36]	0.673
ICU stay in days, median [IQR]	3 [2–5]	3 [3–5]	0.766
Hospital stay in days, median [IQR]	14 [12–25]	15 [12–21]	0.719
12-months biliary complications, n (%)	3 (10.3)	5 (10.9)	0.943

Bold highlights statistical significancy

IQR, interquartile range; SGOT, serum glutamic oxaloacetic transaminase; POD, post-operative day; SGPT, serum glutamic pyruvate transaminase; EAD, early allograft dysfunction; HAT, hepatic artery thrombosis; PGNF, primary graft non-function; ICU, intensive care unit

**Fig. 1:**
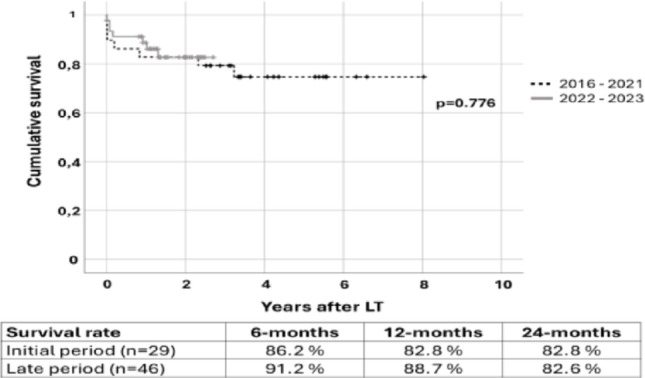
Graft survival curves by period of DCD liver transplant

In terms of survival rates, no significant differences were observed between the two periods (log-rank *p* = 0.776). Survival curves are depicted in Fig. 1.

### Linear correlation analysis

**Fig. 2:**
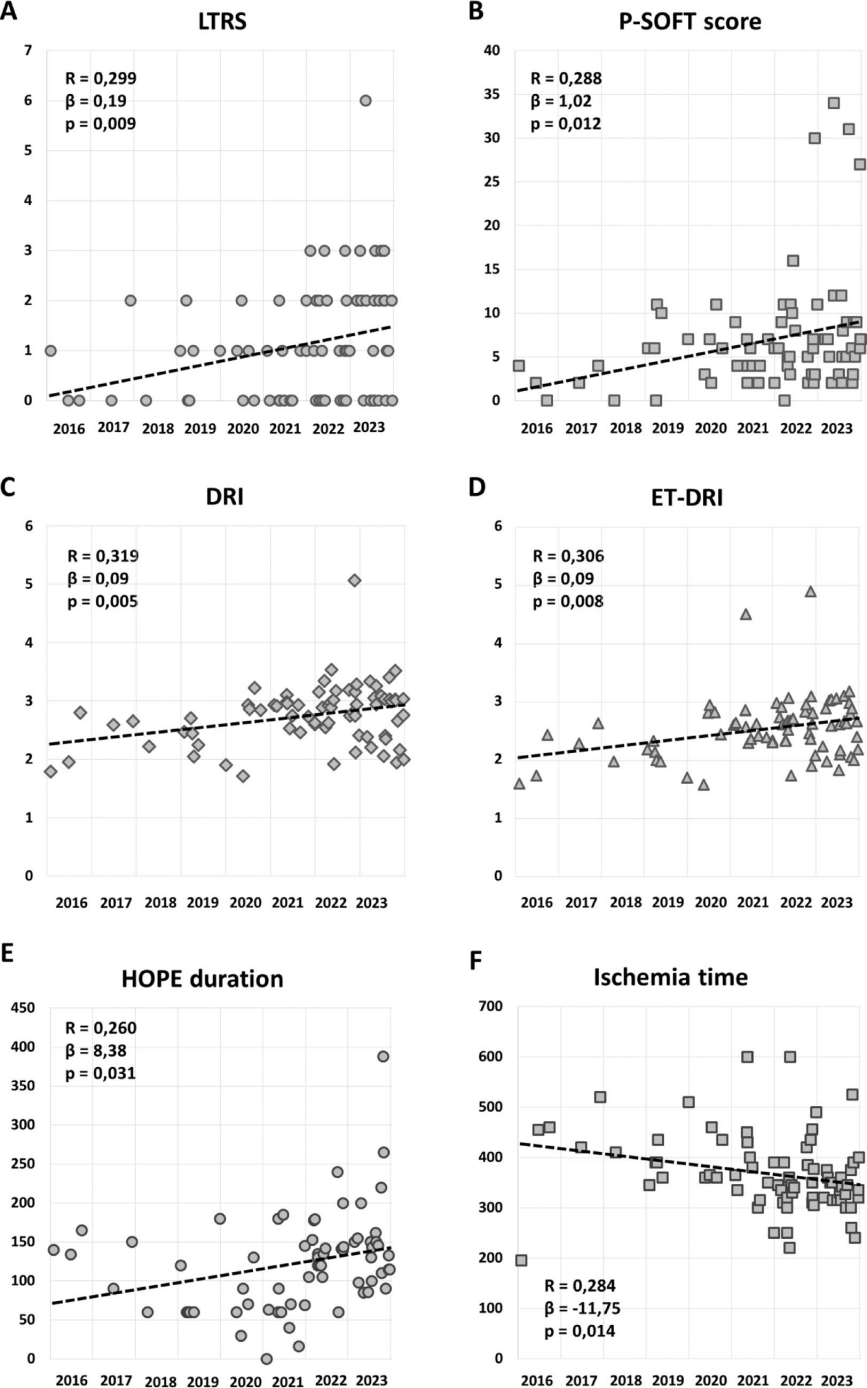
Scatter plots displaying the association between DCD liver transplant date (X-axis) and (**A**) Liver Transplant Risk Score (LTRS), (**B**) Preallocation score to predict Survival Outcomes Following liver Transplantation (P-SOFT), (**C**) Donor Risk Index (DRI), (**D**) EuroTransplant Donor Risk Index (ET-DRI), (**E**) hypothermic oxygenated perfusion (HOPE) duration and (**F**) ischemia time

Linear correlation analysis using the transplant date as independent variable revealed a significant correlation between time since the DCD liver transplant program’s initiation and increasing donor- and recipient-related risk scores (i.e. LTRS, P-SOFT, DRI and ET-DRI). Also HOPE duration and ischemia exhibited a significant correlation: in particular, HOPE duration increased, and ischemia decreased linearly along throughout the study timespan. The results are summarized in Table 4 and illustrated in Fig. 2.

**Table 4:** Linear correlation between time since DCD program’s initiation and donor/recipient complexity parameters as well as allograft reconditioning parameters; all coefficients are calculated per year after DCD program initiation

Variables	β_0_ [95% CI]	β_x_ [95% CI]	*R*	Adjusted *R*^2^	*p*
LTRS	− 0.07 [− 0.98-0.85]	0.19 [0.05–0.33]	0.299	0.077	0.009
P-SOFT	0.49 [− 4.75-5.72]	1.02 [0.23–1.81]	0.288	0.070	0.012
DRI	2.20 [1.79–2.60]	0.09 [0.03–0.15]	0.319	0.089	0.005
ET-DRI	1.98 [1.56–2.41]	0.09 [0.02–0.15]	0.306	0.081	0.008
HOPE duration	70.68 [21.04-120.31]	8.38 [0.78–15.97]	0.260	0.054	0.031
Ischemia time	442.27 [381.16-503.37]	− 11.75 [− 21.00 to − 2.50]	0.284	0.068	0.014

LTRS, Liver Transplant Risk Score; P-SOFT, Preallocation score to predict Survival Outcomes Following liver Transplantation; DRI, Donor Risk Index; ET-DRI, EuroTransplant Donor Risk Index; HOPE, hypothermic oxygenated perfusion.

## Discussion

Over the past decade, advancements in neurocritical care and broader acceptance of organ donation have led to a global increase in DCD organ donors [26]. However, despite the efforts by the transplant community to better understand outcome-determining parameters, optimize allocation strategies, and refine organ recovery practices, the rate of liver transplants has risen more modestly than anticipated, primarily due to limited utilization of DCD donors [26]. Furthermore, contemporary DCD liver transplantation practices exhibit considerable geographical variability in donor management, procurement techniques, recipient selection and utilization of perfusion technologies, resulting in fluctuating rates of organ acceptance, retrieval and implantation across different countries [27, 28]. 

This study provides a comprehensive analysis of the trends and outcomes associated with DCD liver transplantation at a high-volume Italian transplant center over an eight-year period. Our findings align with the global rise in donations after cardiovascular determination of death [3, 26, 27], demonstrating a tenfold increase in the annual rate of DCD liver transplants during the study period (Fig. 1). Moreover, although not included in this study, the year 2024 saw a further increase in DCD liver transplantations, accounting for nearly one-third of all liver transplants performed that year (35/109, 32.1%).

Consistent with the study hypothesis, donor and recipient profiles underwent substantial changes throughout the study period, reflecting accumulated experience and advancements in organ reconditioning. Recipients transplanted in the later period more frequently presented with severe end-stage liver disease (as classified by the Child-Pugh score) and faced a higher risk of perioperative mortality and graft loss, as indicated by LTRS and P-SOFT scores. Additionally, although not statistically significant, DCD allografts were successfully employed in one case of urgent retransplantation and another case of combined liver-kidney transplant during the later period.

Donor characteristics also changed markedly over time. In the later period, the median donor age increased to 72 years – ten years higher than in the initial period. Notably, this phase included a successful DCD liver transplant from an 86-year-old donor. The proportion of ECD and the number of extended criteria per donor also rose significantly, resulting in higher DRI and ET-DRI scores. Histological allograft characteristics evolved as well, with a higher prevalence of Ishak stage 2–3 fibrosis among allografts transplanted in the later period. This finding in our opinion is mainly related to the increased donor age in the later period, being donor age ≥ 65 years the most common feature for donor characterization as ECD in the whole study population and in the later cohort in particular (58.7% vs. 37%, *p* = 0.041).

Interestingly, a considerable proportion of transplants in this cohort would be classified as futile according to the UK DCD risk score. This outcome was anticipated, given that prolonged fWIT (> 30 min) is one of the strongest predictors of graft failure in this scoring system and that Italian legislation highly conditions fWIT duration; in fact, only two cases in the entire study had a fWIT ≤ 30 min [20]. This finding remarks the great potential of NRP for allograft reconditioning in the context of cardiovascular death determination: extracorporeal support in facts allows the utilization of grafts with prolonged fDWIT, whose super-rapid procurement would have otherwise resulted in almost certain transplant failure. The percentage of potentially futile transplants – by means of UK DCD Risk Score – increased in the later period (69.5% vs. 55.2%), although the difference failed to reach statistical significancy.

Despite the progressive increase in donor and recipient risk factors, liver transplant outcomes remained substantially stable between the initial and later period, with comparable rates of organ dysfunction, surgical complications, hospital stay and graft survival. Given the higher marginality of donors in the later period, the preservation of similar allograft functional outcomes was likely influenced by prolonged HOPE reconditioning in this cohort (138 vs. 70 min, *p* < 0.001) and shorter ischemia time (341 vs. 390 min, *p* = 0.002). Notably, both HOPE duration and ischemia time demonstrated a mild linear with the time elapsed since the inception of the DCD program (positive and negative, respectively). The efficacy of prolonged HOPE treatment and ischemia minimization in mitigating ischemia-reperfusion injury is further supported by the lower SGPT flare observed during the first post-transplant week in the later period.

Over the study period, greater confidence in organ perfusion techniques and donation management led to logistical adjustments aimed at minimizing ischemia times and extending HOPE duration. This shift in practice was primarily supported by the findings of Dondossola et al. [29], who demonstrated that prolonged cold ischemia time has detrimental effects on graft function in liver transplantation using marginal grafts, even when reconditioned with HOPE. Notably, the perfusion duration observed in the later period aligns with findings by Eden et al., who recently reported excellent survival rates in a large international cohort of DBD and DCD liver transplants treated with HOPE, supporting its routine implementation in clinical practice [30]. Moreover, although the optimal duration of hypothermic perfusion remains debated, HOPE prolongation has been shown to maintain transplant outcomes while reducing the risk of acute kidney injury, as evidenced by De Carlis et al. [31]

The stability of post-operative complication rates, PGNF, and retransplantation rates across both periods further underscores the feasibility and safety of expanding the DCD donor pool, even under challenging conditions such as the prolonged asystolic periods mandated by the Italian legislation. Notably, the rates of allograft dysfunction and biliary complications observed in this study were significantly lower than those reported in the literature [5, 32], highlighting the potential benefits of sequential normothermic regional perfusion (NRP) and end-ischemic ex situ machine perfusion in reconditioning DCD and EC-DCD liver allografts, as previously documented in the Italian clinical setting [6, 33]. 

The progressive shift towards a less restrictive utilization of DCD liver allografts and the increasing marginality of donors observed in this study suggest that our center’s policies and practices have evolved, implementing allograft reconditioning with accumulated experience. Consequently, donor and recipient risk profiles (i.e. LTRS, P-SOFT score, DRI and ET-DRI) exhibited a mild yet significant linear correlation with the time that elapsed since the inception of the DCD liver transplant program. Similarly, HOPE duration and ischemia time demonstrated a linear correlation with time, reflecting a strategic shift toward prolonged hypothermic perfusion and minimized ischemia, coherently with the findings of Dondossola et al. [29] These findings, together with the increasing severity of recipients’ liver dysfunction and growing marginality of donors, align with other reports highlighting the broader and more versatile use of DCD allografts in liver transplantation over time [3], particularly when organs can be effectively reconditioned to mitigate ischemia-reperfusion injury. Overall, the findings from eight years of DCD liver transplantation practice demonstrate that donation after cardiovascular determination of death has not only expanded the donor pool but has also evolved from an initially perceived marginal practice to a more established and routine source of allografts, whose increasing marginality benefits from routine treatment with sequential NRP and extended end-ischemic HOPE. We hypothesize that the implementation of a more effective reconditioning strategy—particularly with respect to the duration of hypothermic oxygenated perfusion (HOPE)—accounts for the comparability of biliary complication rates and survival outcomes throughout the study period, despite the progressive increase in donor and recipient marginality. Although the one-year survival rate was marginally below 90%, the survival curve was adversely influenced by five early patient deaths unrelated to graft function, which had a disproportionate effect on survival estimates given the limited sample size.

These results show that the progressive expansion of DCD allocation in and Italian-based transplant setting has clearly contributed to increased transplant activity. In particular, the integration of NRP and end-ischemic HOPE has proven effective in offsetting the intrinsic risks associated with prolonged functional warm ischemia, thereby allowing safe expansion toward older and more marginal donors.

### Limitations

The observational nature and monocentric design of this study limit the generalizability of its conclusions, particularly given the unique characteristics of the Italian clinical setting and legislation compared to other countries [6, 28]. Nonetheless, despite the higher technical complexity of donation after cardiovascular determination of death in Italy, Italian DCD liver transplants have demonstrated comparable outcomes when included in international multicenter case series [8, 28, 30]. Additionally, the study design resulted in a limited follow-up period for patients in the later cohort, although a minimum follow-up of twelve months was ensured for all participants.

### Conclusions

This study highlights the significant evolution of DCD liver transplantation practices at our center, demonstrating a linear correlation between time since program inception and increasing donor and recipient complexity. Furthermore, the stability of transplant outcomes despite growing donor- and recipient-related risks highlights that DCD liver allografts are more versatile and less marginal than initially feared. As global DCD liver transplantation practices continue to exhibit significant geographical variability, evaluating DCD donor potential in diverse clinical settings can challenge historical perceptions of their marginality. In this sense, NRP and end-ischemic HOPE represent a valuable and effective strategy for reconditioning DCD allografts, enhancing their utilization in high-risk donor and recipient scenarios. Future research should focus on refining risk stratification and exploring novel preservation technologies to optimize high-risk DCD donor utilization. Additionally, long-term multicenter studies could further validate these findings across diverse clinical settings and guide the development of standardized protocols for DCD donor optimization worldwide.

## Supplementary Information

Below is the link to the electronic supplementary material.

Supplementary Material 1: Liver transplants performed at Policlinico Sant’Orsola – IRCCS Azienda Ospedaliero-Universitaria di Bologna by donor type per year.

## Data Availability

The data that support the findings of this study are available from the corresponding author [MR], upon reasonable request.

## Abbreviations

CCI®: Comprehensive Complication Index®
DBD: Donation/donor after brainstem death
DCD: Donation/donor after cardiovascular determination of death
DRI: Donor Risk Index
DWIT: Donor warm ischemia time
fDWIT: Functional donor warm ischemia time
EAD: Early allograft dysfunction
ECD: Extended criteria donor
EC-DCD: Extended criteria donor after cardiovascular determination of death
ET-DRI: EuroTransplant Donor Risk Index
HAT: Hepatic artery thrombosis
HOPE: Hypothermic oxygenated perfusion
ICU: Intensive care unit
LT: Liver transplantation
LTRS: Liver Transplant Risk Score
NRP: Normothermic regional perfusion
PGNF: Primary graft non function
P-SOFT: Preallocation score to predict Survival Outcomes Following liver Transplantation
SCS: Static cold storage
SGOT: Serum glutamic oxaloacetic transaminase
SGPT: Serum glutamic pyruvic transaminase
URL: Upper reference limit
