# Supplementary figures and images for: A journey from marginality to routine and beyond: single center experience with DCD utilization for liver transplantation in Italy
Source: Updates Surg. 2026 Mar 10;78(4):1695–704. doi: 10.1007/s13304-026-02581-2 (PMC13421298; doi:10.1007/s13304-026-02581-2)

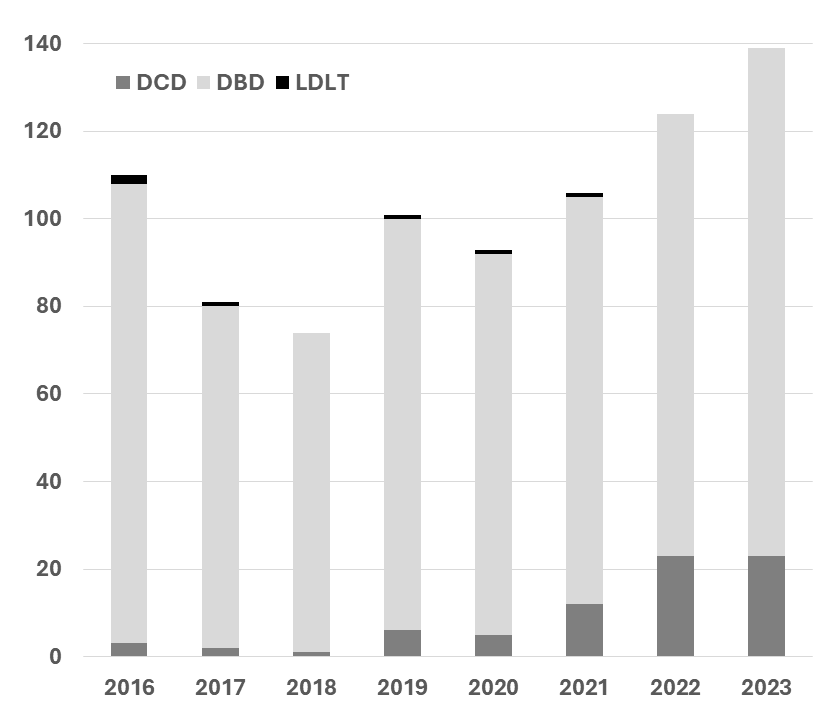

Supplement: Supplementary file 1 — Supplementary Material 1: Liver transplants performed at Policlinico Sant’Orsola – IRCCS Azienda Ospedaliero-Universitaria di Bologna by donor type per year. [file 13304_2026_2581_MOESM1_ESM.tiff]
